# Sequential Cranial Ultrasound and Cerebellar Diffusion Weighted Imaging Contribute to the Early Prognosis of Neurodevelopmental Outcome in Preterm Infants

**DOI:** 10.1371/journal.pone.0109556

**Published:** 2014-10-20

**Authors:** Margaretha J. Brouwer, Britt J. M. van Kooij, Ingrid C. van Haastert, Corine Koopman-Esseboom, Floris Groenendaal, Linda S. de Vries, Manon J. N. L. Benders

**Affiliations:** 1 Department of Neonatology, Wilhelmina Children’s Hospital/University Medical Center Utrecht, Utrecht, The Netherlands; 2 Centre for the Developing Brain, Division of Imaging Sciences and Biomedical Engineering, King's College London, St Thomas' Hospital, London, United Kingdom; Robert Debre Hospital, France

## Abstract

**Objective:**

To evaluate the contribution of sequential cranial ultrasound (cUS) and term-equivalent age magnetic resonance imaging (TEA-MRI) including diffusion weighted imaging (DWI) to the early prognosis of neurodevelopmental outcome in a cohort of very preterm infants (gestational age [GA] <31 weeks).

**Study design:**

In total, 93 preterm infants (median [range] GA in weeks: 28.3 [25.0–30.9]) were enrolled in this prospective cohort study and underwent early and term cUS as well as TEA-MRI including DWI. Early cUS abnormalities were classified as normal, mild, moderate or severe. Term cUS was evaluated for ex-vacuo ventriculomegaly (VM) and enlargement of the extracerebral cerebrospinal fluid (eCSF) space. Abnormalities on T1- and T2-weighted TEA-MRI were scored according to Kidokoro et al. Using DWI at TEA, apparent diffusion coefficients (ADCs) were measured in four white matter regions bilaterally and both cerebellar hemispheres. Neurodevelopmental outcome was assessed at two years’ corrected age (CA) using the Bayley Scales of Infant and Toddler Development, third edition. Linear regression analysis was conducted to explore the correlation between the different neuroimaging modalities and outcome.

**Results:**

Moderate/severe abnormalities on early cUS, ex-vacuo VM and enlargement of the eCSF space on term cUS and increased cerebellar ADC values on term DWI were independently associated with worse motor outcome (*p*<.05). Ex-vacuo VM on term cUS was also related to worse cognitive performance at two years’ CA (*p*<.01).

**Conclusion:**

These data support the clinical value of sequential cUS and recommend repeating cUS at TEA. In particular, assessment of moderate/severe early cUS abnormalities and ex-vacuo VM on term cUS provides important prognostic information. Cerebellar ADC values may further aid in the prognostication of gross motor function.

## Introduction

During the last decades, several neuroimaging modalities have been introduced to evaluate the preterm brain during the neonatal period in order to improve prediction of developmental impairments.

Cranial ultrasound (cUS) has proven to be an easily accessible neuroimaging technique, which can be performed at the bedside to detect a variety of brain lesions in the preterm infant. Significant associations have been reported between severe cUS abnormalities, such as germinal matrix-intraventricular hemorrhage (GMH-IVH) grade III or IV according to Papile et al. [1] or cystic periventricular leukomalacia (PVL) according to de Vries et al. [2], and an unfavourable cognitive and motor outcome. [3] Conventional magnetic resonance imaging performed around term-equivalent age (TEA-MRI) or even soon after birth has been shown to be superior to cUS in detecting more subtle brain lesions. [4] Scoring systems have been developed for white matter (WM) and gray matter (GM) abnormalities in order to better predict the risk of an adverse outcome. [5] An adjusted brain scoring system for TEA-MRI has recently been published, which also incorporates evaluation of the cerebellum [6], reflecting the increased recognition of the impact of cerebellar abnormalities on neurodevelopment [7], [8].

The application of diffusion weighted imaging (DWI) was introduced to assess the microstructural development of WM in the preterm brain. [9] Data on the correlation between WM apparent diffusion coefficients (ADCs) and brain injury [10]–[13] or neurodevelopmental outcome [14]–[16] remain, however, controversial. A few studies have addressed the effect of supratentorial brain injury on cerebellar ADC values, with inconclusive results. [12], [17] No data are available on the prognostic value of cerebellar ADC values.

With the increasing availability of neuroimaging modalities, the question arises to which extent they are complementary, at which point in time they are most predictive and whether TEA-MRI should be performed in every extremely low birth weight infant. The aim of this cohort study among very preterm infants was to examine the contribution of sequential cUS and TEA-MRI including DWI to the early prognosis of neurodevelopmental outcome at two years’ corrected age (CA).

## Materials and Methods

### Patients

From October 2006 to March 2008, very preterm infants (gestational age [GA] <31 weeks) admitted to the level three unit of the Wilhelmina Children’s Hospital, Utrecht, The Netherlands, were recruited for a prospective neuroimaging study approved by the Medical Ethics Committee of our institute. Neonates who were born outside our referral district or with congenital anomalies were excluded. Of the 168 consecutively admitted neonates, eighteen infants deceased before reaching TEA, no parental informed consent was obtained for fifteen infants and sixteen infants were examined on a 1.5 Tesla system, resulting in 119 infants with 3.0 Tesla MR imaging at TEA. From this cohort, we further excluded infants with congenital brain abnormalities on sequential cUS and TEA-MRI (*n* = 2; i.e., heterotopia, antenatal porencephalic cyst) as well as infants who were scanned at a postmenstrual age (PMA) ≥44 weeks (*n* = 2) or with motion artifacts on DWI (*n* = 18). Four infants were lost to follow up at two years’ CA, leaving 93 infants eligible for final inclusion. Written parental informed consent was obtained for all infants.

### Neuroimaging

#### Early and term cUS

Ultrasound scans were performed within six hours of admission, at least three times in the first week after birth and then weekly till discharge to a level two hospital. These scans are referred to as *early cUS*. At TEA, within 24 hours following MRI, cUS was repeated. This scan is referred to as *term cUS*. Scanning was performed with a Toshiba Aplio (Toshiba Medical Systems, Zoetermeer, The Netherlands) or ATL-5000 ultrasound machine (Philips Medical Systems, Best, The Netherlands) with a transducer frequency of 5–8 MHz. Using the anterior fontanel as an acoustic window, standard views were taken in the coronal and sagittal planes. Comprehensive evaluation of the cerebellum through the mastoid and posterior fontanel was not performed at the time in all neonates. Hence, cerebellar cUS abnormalities could not be analyzed in this paper.

#### TEA-MRI

MR images were acquired on a 3.0 Tesla MR system (Philips Healthcare, Best, The Netherlands) using a sense head coil. Infants were sedated with 50–60 mg/kg chloralhydrate fifteen minutes prior to the examination. The protocol involved sagittal T1-weighted imaging (repetition time [TR] = 886 ms; echo time [TE] = 15 ms; slice thickness = 3.0 mm), axial 3DT1-weighted imaging (TR = 9.4 ms; TE = 4.6 ms; slice thickness = 2.0 mm, no gap), axial T2-weighted imaging (TR = 6293 ms; TE = 120 ms; slice thickness = 2.0 mm, no gap) and axial DWI (TR = 2407 ms; TE = 68 ms; voxel size = 0.9×0.9×4.0 mm; b-values = 0 and 800 mm^2^/s).

### Assessment of brain injury

#### Early cUS

Assessment of brain injury was performed offline by two authors (MJNLB, LSV) using DicomWorks 1.3.5 (www.dicomworks.com). For each infant, a final classification was applied to the whole set of early cUS scans based on the most severe lesion observed. Early cUS findings were classified as 1) normal when no or just minor abnormalities were observed (e.g., germinal layer cysts, plexus cysts, subependymal pseudocysts and/or lenticulostriate vasculopathy); 2) mildly abnormal in the presence of a GMH-IVH grade I or II according to Papile et al. [1], mild subsequent ventricular dilatation and/or PVL grade I according to de Vries et al. [2]; 3) moderately abnormal if a GMH-IVH grade III was diagnosed and 4) severely abnormal in case of a GMH-IVH grade IV, progressive post-haemorrhagic ventricular dilatation (PHVD, i.e., ventricular index >97^th^ percentile according to Levene [18], anterior horn width >6 mm [19] and/or thalamo-occipital distance >24 mm [19]) requiring intervention, cystic PVL grade II or III, parenchymal lobar haemorrhage and/or focal infarction.

#### Term cUS

**Figure 1:**
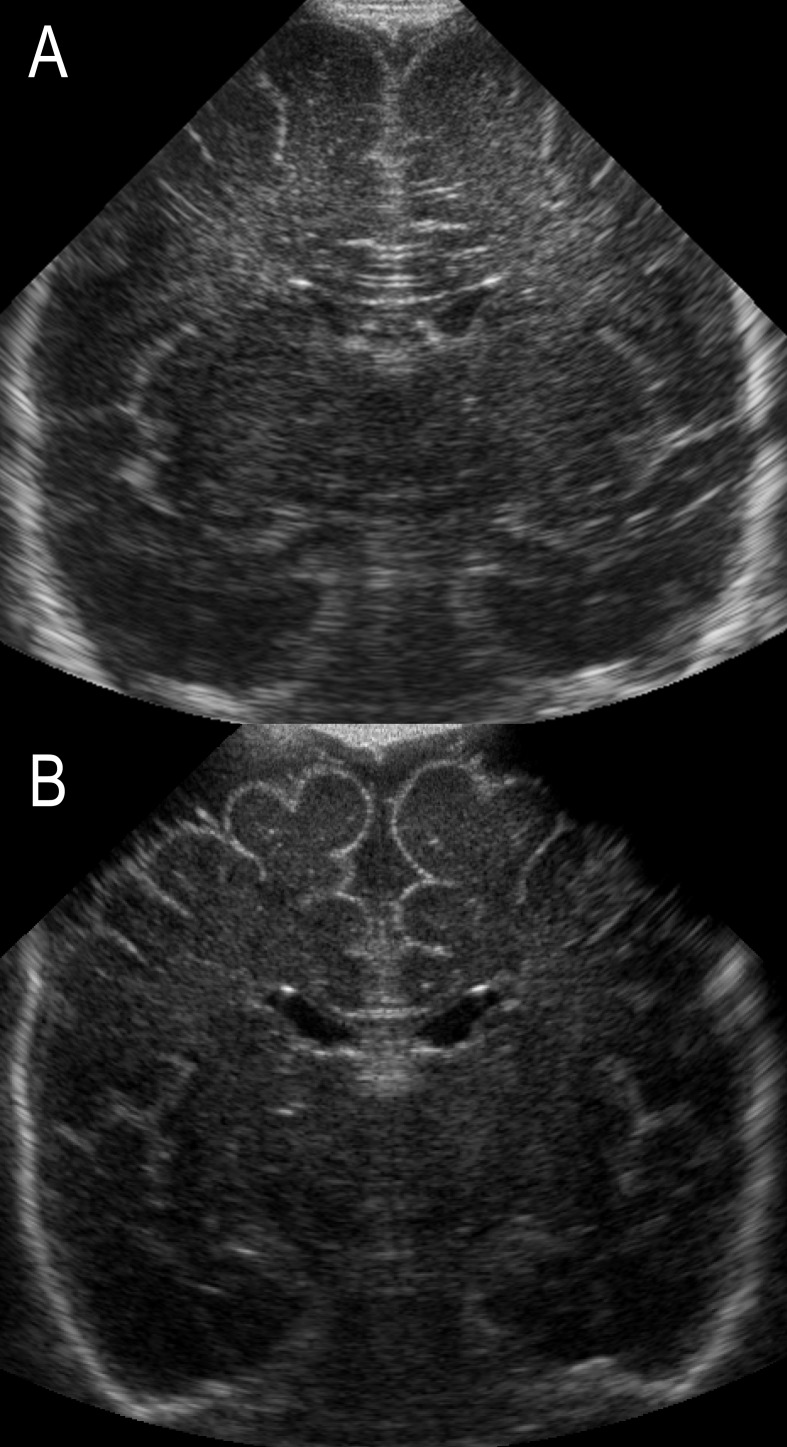
Term cUS of two preterm born infants; coronal views with (A) normal findings and (B) enlargement of the eCSF space with an increase in interhemispheric distance and subarachnoid space.

**Figure 2:**
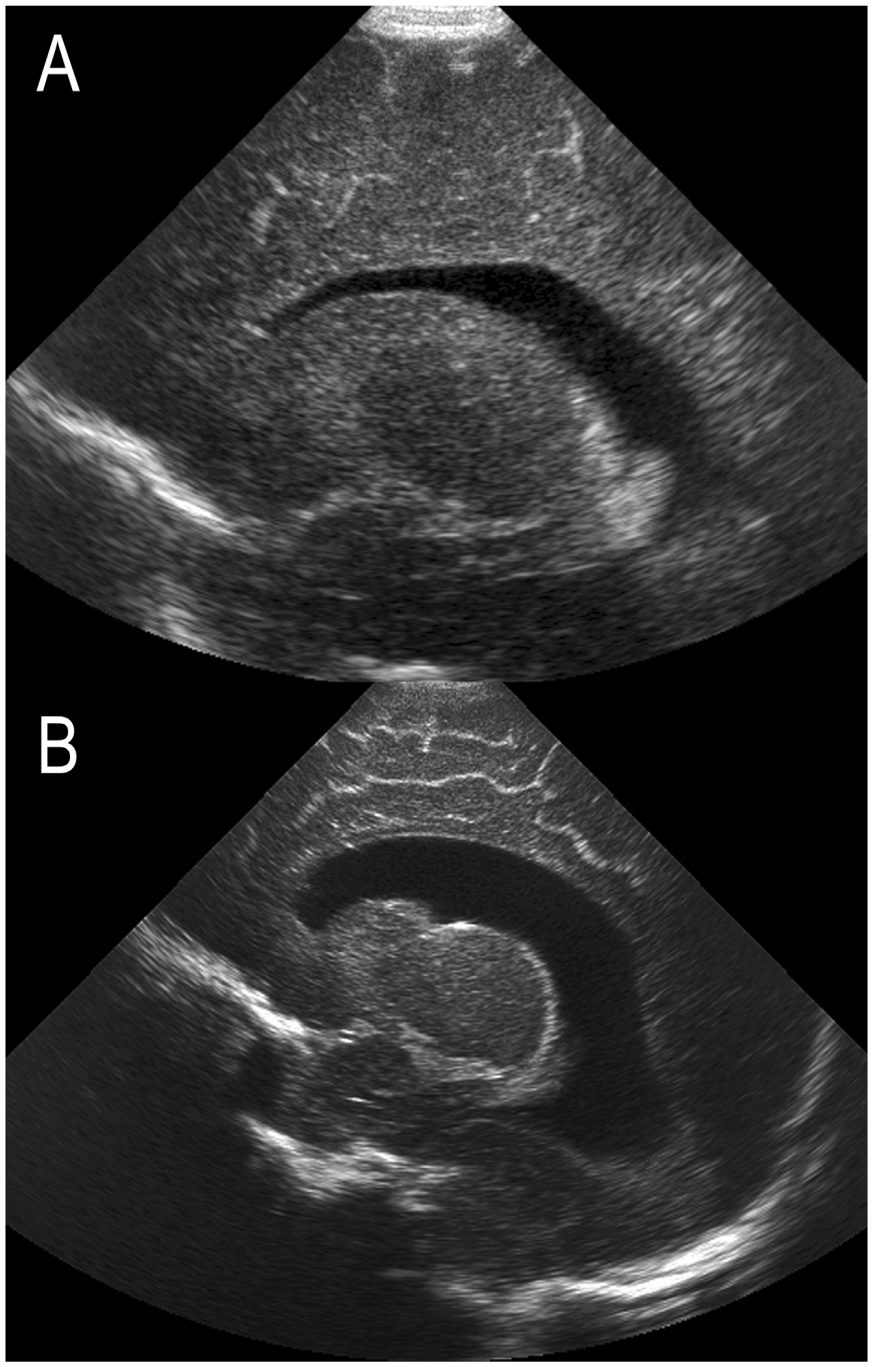
Term cUS of two preterm born infants; parasagittal views (A) following diffuse WM injury without prior GMH-IVH, referred to as ex-vacuo VM, and (B) following a GMH-IVH grade II and subsequent PHVD requiring intervention. Note the posterior dilatation and irregular ventricular margins in (A), in contrast to the more global ventricular enlargement in (B).

Term cUS scans were qualitatively scored by one of the authors (LSV) for the presence of 1) enlargement of the extracerebral cerebrospinal fluid (eCSF) space, defined as an increase in interhemispheric distance and/or subarachnoid space (Figure 1) [20] and 2) ex-vacuo ventriculomegaly (VM), defined as an increase in ventricular size – often most pronounced for the occipital horns – with irregularity of the ventricular margins (Figure 2A). [21] Ex-vacuo VM, considered as a sequel of WM injury, was distinguished from PHVD on the basis of ventricular shape. In contrast to ex-vacuo VM, PHVD tends to be associated with so-called “ballooning” of the ventricles with both anterior and posterior dilatation without an irregular ventricular shape (Figure 2B). [21] In infants with prior GMH-IVH, early cUS did further aid in the discrimination between these two entities.

#### T1- and T2-weighted TEA-MRI

WM, cortical and deep GM and cerebellum were evaluated for the presence of brain abnormalities (assessed by LSV) and abnormal brain metrics (measured by MJB) according to the scoring system by Kidokoro et al. [6] for T1- and T2-weighted TEA-MRI. Measurements were obtained using OsiriX (32-bit version, www.osirix-viewer.com), which allowed for free conversion to all planes. A global brain abnormality score was calculated as the sum of these regional scores and further classified as normal (0–≤3), mildly abnormal (4–≤7), moderately abnormal (8–≤11) and severely abnormal (≥12) according to Kidokoro et al [6].

#### Term DWI – ADC values

**Figure 3:**
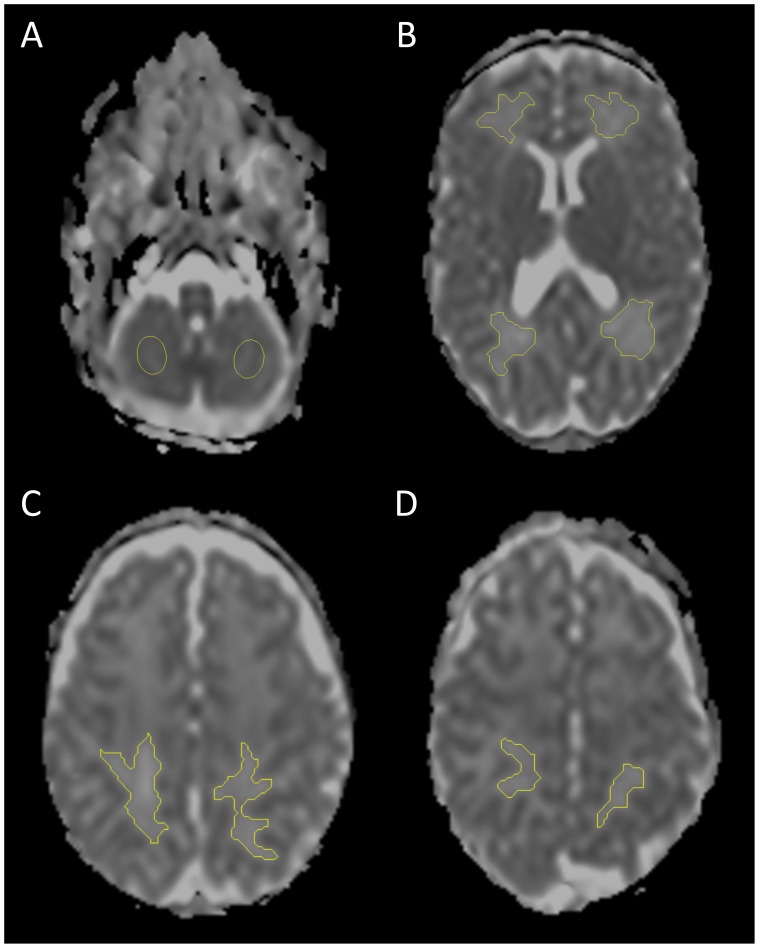
Evaluation of ADC values on term DWI in ten ROIs in (A) cerebellum and (B) frontal, occipital, (C) parieto-occipital and (D) parietal WM.

ADC values were measured in ten regions of interest (ROIs) in the WM and cerebellum (Figure 3). Measurements were performed by two independent observers (MJB, BJMK) using a standalone MR work station (Achieva, Philips Medical Systems, Best, The Netherlands).

For the WM, ADC maps and ADC maps with enhanced contrast (*e*-ADC) were first derived from the diffusion weighted images. ROIs were then manually drawn on the *e*-ADC map using a *seed growing tool* and copied to the ADC map. The frontal and occipital WM were assessed at the level of the foramen of Monro and the trigone, respectively. Sagittal T1-weighted MRI was used to define the cross-sectional planes for ADC measurements of the parieto-occipital and parietal WM, in general two and three slices above the level of the trigone, respectively. Care was taken to avoid partial volume averaging from CSF or cortical GM on the slices above and below. Cerebellar ROIs were positioned at the level of the maximal cerebellar hemispheric width. Given the complex structure of the cerebellum and partial volume effects, large ROIs were placed in the center of both hemispheres instead of selecting a particular tissue type. In infants with profound supra- or infratentorial parenchymal lesions, ADC measurements were not performed in the areas involved.

For nineteen infants selected at random, ADC measurements were repeated by both observers after a time interval of at least 24 hours. After calculating the intra- and interobserver reliability, both observers’ ADC measurements were averaged. Means were calculated for bilateral ADC measurements of each WM region and the cerebellum and used in consecutive analyses.

### Neurodevelopmental outcome

Neurodevelopmental outcome was assessed at 24 months’ CA by a single developmental specialist (ICH) using the cognitive, fine motor and gross motor subtest of the Bayley Scales of Infant and Toddler Development, third edition (BSITD-III). The composite and scaled scores corrected for premature birth were calculated (mean [SD] in a normative population: 100 [15] and 10 [3], respectively).

### Statistical analysis

Data were analyzed using IBM SPSS Statistics version 20 (SPSS Inc, Chicago, Illinois).

The intra- and interobserver reliability for ADC measurements were assessed by calculating the intraclass correlation coefficients for single measures using the two-way random model for absolute agreement.

To evaluate the relationship between the different neuroimaging modalities, several statistical tests were conducted. Categorical variables were analyzed using either a Chi-square or Fisher’s exact test. The distribution of continuous variables between groups was compared using ANOVA and Mann-Whitney *U* tests for parametric and non-parametric data, respectively. Continuous data were compared by calculating a Spearman’s rho. The distribution of GMH-IVH and PHVD among infants with and without ex-vacuo VM on term cUS was compared using a Chi-square and Fisher’s exact test, respectively.

The correlation between neuroimaging and outcome was evaluated using linear regression analysis with outcome as dependent variable. Cognitive and fine motor outcome were corrected for maternal education; gross motor outcome was not related to maternal education. First, the association between the different neuroimaging modalities and outcome was explored. Next, neuroimaging parameters were combined into one model. This was done by hand in a forward manner; entrance criterion was a *p-*value <.10, exclusion criterion a *p-*value ≥.05. Finally, the additional influence of gender, GA and birth weight z-score on outcome was assessed.

## Results

### Descriptive results

A total number of 93 preterm neonates (50 males, 43 females) were enrolled (median [range] GA in weeks: 28.3 [25.0–30.9]; birth weight in grams: 1060 [630–1910]; birth weight z-score: 0.05 [−1.14–2.06]). The maternal educational level was low, intermediate and high for, respectively, 23 (24.7%), 38 (40.6%) and 30 (32.3%) infants; data for two infants were missing. TEA-MRI was performed at a median (range) PMA of 41.6 (39.6–43.6) weeks. Neurodevelopmental assessment (BSITD-III) was conducted at a median (range) CA of 24.1 (23.6–27.6) months. The infants’ neuroimaging findings and neurodevelopmental outcome are summarized in Table 1.

**Table 1:** Descriptive results (*n* = 93).

**Early cUS; ** ***n*** ** (%)**		
Normal		26 (28.0)
Mild abnormalities		55 (59.1)
Moderate abnormalities		3 (3.2)
Severe abnormalities		9 (9.7)
**Term cUS; ** ***n*** ** (%)** $		
Ex-vacuo VM		18 (19.4)
Enlargement eCSF space		41 (44.1)
**T1- and T2-weighted TEA-MRI; median (IQR; range)**		
Global brain abnormality score		3 (2–6; 0–15)
WM score		3 (2–4; 0–11)
Cortical GM score		0 (0; 0–3)
Deep GM score		0 (0; 0–3)
Cerebellum score		0 (0–1; 0–7)
Term DWI – ADC values *10 ^−**3**^ ** mm^2^**/**s; median (IQR; range)** Δ		
Frontal WM	R	1.611 (1.516–1.666; 1.292–1.840)
	L	1.626 (1.526–1.679; 1.324–1.837)
Occipital WM	R	1.579 (1.508–1.661; 1.367–1.794)
	L	1.607 (1.523–1.687; 1.356–1.843)
Parieto-occipital WM	R	1.654 (1.581–1.716; 1.409–1.843)
	L	1.662 (1.579–1.727; 1.434–1.870)
Parietal WM	R	1.610 (1.538–1.671; 1.382–1.923)
	L	1.600 (1.524–1.664; 1.408–1.906)
Cerebellum	R	1.075 (1.021–1.121;.920–1.242)
	L	1.078 (1.025–1.125;.911–1.271)
**Neurodevelopmental outcome (BSITD-III) at 24 months’ CA; median (IQR; range)**		
Cognitive composite score		105 (95–110; 70–145)
	Cognitive scaled score	11 (9–12; 4–19)
Motor composite score		107 (100–112; 73–142)
	Fine motor scaled score	13 (11–14; 5–19)
	Gross motor scaled score	9 (9–10; 6–15)

IQR: interquartile range; L: left hemisphere; R: right hemisphere;

$ term cUS data were missing for ten infants;

Δ presented are ADC values uncorrected for PMA.

#### Early cUS

Since only twelve neonates (12.9%) demonstrated moderate or severe abnormalities on early cUS, they were combined into one group. Infants with no (*n* = 26, 28.0%) or mild (*n* = 55, 59.1%) cUS abnormalities did not differ with respect to neuroimaging findings at TEA or outcome at two years’ CA and were therefore also analyzed together.

#### Term cUS

Ex-vacuo VM at TEA was not related to a previous GMH-IVH and/or PHVD. Three out of 18 infants (16.7%) with ex-vacuo VM previously suffered from a severe GMH-IVH (i.e., two infants with a GMH-IVH grade III and one infant with a small unilateral venous infarction) and subsequently developed PHVD requiring CSF drainage. At TEA, these infants demonstrated bilateral irregularity of the ventricular margins, suggestive of ex-vacuo dilatation. Among the infants without ex-vacuo VM on term cUS, three out of 65 infants (4.6%; term cUS data for ten infants were missing) were previously diagnosed with a severe GMH-IVH and developed PHVD requiring intervention (*p* = .113).

No association was observed between ex-vacuo VM and enlargement of the eCSF space on term cUS.

#### T1- and T2-weighted TEA-MRI

Eight infants (8.6%) infants were classified with moderate/severe brain abnormalities on TEA-MRI; the remaining 85 infants (91.4%) were classified as having no or just mild brain abnormalities.

#### Term DWI – ADC values

A high intra- and interobserver reliability (≥0.81) could be achieved according to the strength of agreement scale by Brennan and Silman [22] for ADC measurements in all ten assessed ROIs.

ADC values showed a downward trend with increasing PMA in all WM regions (*p*<.10) and in the cerebellum (*p*<.001), irrespective of the presence of brain injury on sequential cUS or TEA-MRI. The mean (95% confidence interval, CI) decrease in ADC per week PMA ranged from −.018 (−.039;.003) to −.033 (−.055; −.011) * 10^−3^ ****mm^2^/s for the different WM regions and was −.038 (−.050; −.026) * 10^−3^ ****mm^2^/s for the cerebellum. Hence, ADC values were corrected for PMA using linear regression analysis (i.e., corrected ADC = measured ADC+the slope * [PMA −40]).

### Relationship between neuroimaging modalities

No statistically significant association could be demonstrated between moderate/severe early cUS abnormalities and enlargement of the eCSF space or ex-vacuo VM on term cUS.

Both moderate/severe abnormalities on early cUS and ex-vacuo VM on term cUS were associated with higher global brain abnormality scores on TEA-MRI (*p*<.01; Table 2). The majority of infants with no/mild early cUS abnormalities (79/81, 97.5%) had a normal or just mildly abnormal TEA-MRI scan according to Kidokoro et al. [6]; the remaining two infants (2.5%) demonstrated diffuse WM and cerebellar injury on TEA-MRI, classified as moderately abnormal. Among the twelve infants with moderate/severe early cUS abnormalities, six infants (50%) were classified as having a normal or just mildly abnormal TEA-MRI. Five of these infants previously suffered from a GMH-IVH grade III, four of whom subsequently developed PHVD requiring intervention; one infant had a parenchymal hemorrhage which revolved into a single large cyst.

**Table 2:** Comparison between sequential cUS and T1- and T2-weighted TEA-MRI (*n* = 93).

	Early cUS			Term cUS$			Term cUS$		
	*Normal/Mild*	*Moderate/Severe*		*Ex-vacuo VM*			*Enlargement eCSF space*		
				*No*	*Yes*		*No*	*Yes*	
	*(n = 81)*	*(n = 12)*	*P*	*(n = 65)*	*(n = 18)*	*p*	*(n = 42)*	*(n = 41)*	*p*
**TEA-MRI; median (range)**									
Global brain abnormality score	3 (0–9)	7.5 (3–15)	.000	3 (0–13)	5.5 (2–15)	.003	3 (0–15)	3 (1–13)	.963
WM score	2 (0–7)	6 (3–11)	.000	2 (0–11)	4 (1–8)	.003	3 (0–8)	3 (0–11)	.864
Cortical GM score	0 (0–3)	0 (0–1)	.903	0 (0–2)	0 (0–3)	.195	0 (0–1)	0 (0–3)	.001
Deep GM score	0 (0–1)	0 (0–3)	.022	0 (0–1)	0 (0–3)	.153	0 (0–3)	0 (0–1)	.314
Cerebellum score	0 (0–3)	2 (0–7)	.001	0 (0–7)	0 (0–7)	.438	0 (0–7)	0 (0–2)	.015

$ term cUS data were missing for ten infants.

Neither early and term cUS findings nor global brain abnormality scores or the different subscores on TEA-MRI were related to ADC values of the WM or cerebellum on term DWI.

### Neuroimaging versus outcome

**Cognitive outcome.** Ex-vacuo VM on term cUS was associated with worse cognitive outcome at two years’ CA (β in composite scores [95% CI]: −9.2 [−14.6; −3.7], *p* = .001). A trend towards worse cognitive performance was observed for moderate/severe abnormalities on early cUS (−6.2 [−13.5; 1.1], *p* = .094) and global brain abnormality scores on TEA-MRI (−.8 [−1.6;.0], *p* = .061). When combined in the final model, only ex-vacuo VM on term cUS remained statistically significant (Table 3). The negative association between ex-vacuo VM and cognitive outcome remained unmodified after correcting for a preceding GMH-IVH or PHVD. Adding gender, GA or birth weight z-score did not significantly improve the model.

**Table 3:** Table **3.** Correlation between the different neuroimaging modalities and neurodevelopmental outcome at two years’ CA; presented are the changes in composite and scaled scores for cognitive and motor outcome, respectively, according to the BSITD-III (*n* = 93).

		Multilinear regression model			
		*β*	95% CI	*p*	*R^2^*
**Cognitive outcome; composite score (mean [SD]** * **: 100 ** [15] **)**					**.** ***268***
Maternal education		5.9	2.9; 8.8	.000	
Term cUS:	Ex-vacuo VM	−9.2	−14.6; −3.7	.001	
**Fine motor outcome; scaled score (mean [SD]** * **: 10 ** [3] **)**					**.** ***339***
Maternal education		.8	.1; 1.5	.023	
Birth weight z-score		1.2	.5; 1.9	.001	
Early cUS:	Moderate/severe abnormalities	−2.8	−4.6; −1.0	.002	
Term cUS:	Ex-vacuo VM	−2.3	−3.6; −1.1	.000	
**Gross motor outcome; scaled score (mean [SD]** * **: 10 ** [3] **)**					**.** ***364***
Birth weight z-score		.6	.2; 1.1	.008	
Early cUS:	Moderate/severe abnormalities	−2.3	−3.7; −1.0	.001	
Term cUS:	Ex-vacuo VM	−1.6	−2.4; −.7	.001	
	Enlargement eCSF space	−.9	−1.6; −.1	.020	
Term DWI:	Cerebellum ADCΔ	−.6	−1.0; −.2	.003	

*i.e., in a normative population;

Δ presented are the changes in scaled scores per increase in ADC of.100*10^−3^ ****mm^2^/s.

In contrast to qualitative assessment of ex-vacuo VM on term cUS, classification of ventricular size on TEA-MRI as incorporated in the global brain abnormality score by Kidokoro et al. [6] – i.e., based on absolute measurements of atrial size – did not correlate with cognitive outcome nor with fine or gross motor outcome.

#### Fine motor outcome

Ex-vacuo VM on term cUS (β in scaled scores [95% CI]: −2.2 [−3.5; −.8], *p* = .002) and global brain abnormality scores on TEA-MRI (−.3 [−.5; −.1], *p* = .005) were associated with worse fine motor outcome at two years’ CA, whereas a trend was observed for moderate/severe abnormalities on early cUS (−1.5 [−3.2;.2], *p* = .075). When combined, TEA-MRI did not significantly contribute to early and term cUS in predicting fine motor outcome. Adding birth weight z-score further improved the final model, in contrast to GA and gender (Table 3).

#### Gross motor outcome

Moderate/severe abnormalities on early cUS (β in scaled scores [95% CI]: −1.7 [−2.8; −.5], *p* = .004), ex-vacuo VM (−1.4 [−2.4; −.5], *p* = .003) and enlargement of the eCSF space on term cUS (−.9 [−1.7; −.1], *p* = .033), global brain abnormality scores on TEA-MRI (−.2 [−.4; −.1], *p* = .001) as well as cerebellar ADC values on term DWI (−.5 [−.9; −.1], *p* = .026) were all associated with gross motor performance at two years’ CA. When combined, global brain abnormality scores on TEA-MRI did not significantly contribute to sequential cUS and cerebellar ADC values on term DWI in the prediction of gross motor function. Adding birth weight z-score further improved the final model, whereas GA and gender did not contribute (Table 3).

## Discussion

In this study, ex-vacuo VM on term cUS was identified as an independent marker for worse cognitive and motor performance in preterm infants at two years’ CA. Early cUS, assessment of the eCSF space on term cUS and cerebellar ADC values on term DWI further added to the early prognosis of motor outcome in preterm infants.

Moderate/severe early cUS abnormalities were associated with fine and gross motor outcome in agreement with previous data. [3], [21] No correlation with cognitive outcome was observed at two years’ CA; the number of infants with moderate/severe injury on early cUS in our cohort was, however, small.

Ex-vacuo VM on term cUS was related to cognitive, fine motor and gross motor outcome, irrespective of a preceding GMH-IVH and/or PHVD or the presence of moderate/severe brain pathology on early cUS. Ex-vacuo dilatation of the ventricles seems closely intertwined with prematurity. [23], [24] In preterm infants, it has been postulated to reflect a sequel of diffuse WM damage with injury to pre-oligodendrocytes as a key finding. [25] VM has been demonstrated to be more pronounced for the occipital horns and associated with volume loss of the adjacent WM and subcortical GM. [26], [27] In previous studies, VM was shown to pose an additional risk for cognitive, motor and visuomotor impairments as well as behavioural problems, but only in the presence of concomitant brain injury.[28]–[30] Considering ventricular shape in addition to size, though more subjective, may yield more prognostic information according to our data, probably by better identification of infants with ex-vacuo VM. Differentiation between ex-vacuo VM and PHVD is essential but may be difficult, especially in the absence of sequential early cUS as well as in infants with a previous GMH-IVH. Irregular ventricular margins and predominant dilatation of the occipital horns may serve as hallmark of ex-vacuo VM. PHVD, in contrast, is often associated with prior “ballooning” of the ventricles and dilatation of the anterior as well as the posterior part of the ventricles [21].

Enlargement of the eCSF space was a common sonographic finding at TEA and found to be associated with worse gross motor function. Horsch et al. [20] previously identified enlargement of the eCSF space in combination with a reduction in complex gyral folding as a marker for adverse cognitive and motor outcome in very preterm infants. No correction was, however, applied for associated VM or concomitant brain pathology.

The additional prognostic value of T1- and T2-weighted TEA-MRI over sequential high-quality cUS appeared to be limited in our preterm cohort. This may be due to only a small number of infants with severe brain injury and an overall relatively favorable neurodevelopmental outcome. Hence, the prognostic significance of the new MRI scoring system by Kidokoro et al. [6] needs to be reassessed in a larger preterm cohort. Although our data demonstrated that sequential cUS during the neonatal period can reliably predict the absence of major TEA-MRI abnormalities, MRI is known to be superior to cUS in detecting more subtle types of injury, such as punctate WM lesions [4] or small cerebellar hemorrhages [31]. Whereas not necessarily associated with unfavorable neurodevelopment at early age [32], [33], the long term clinical correlates of these brain lesions are still not known.

Cerebellar ADC values on term DWI were found to be associated with gross motor outcome in this study. We hypothesize that increased ADC values may reflect delayed or impaired cerebellar development. This assumption is supported by the observation that cerebellar ADC values decreased with PMA, which might relate to the ongoing increase in size and cell density of particularly the molecular and internal granular layers at the time, hence restricting diffusion. There is growing evidence that supratentorial brain pathology plays a crucial role in the causal pathway of cerebellar underdevelopment, resulting in smaller cerebellar volumes. [8], [34], [35] Reduced cerebellar volumes, as well as the cerebellar N-acetylaspartate/choline ratio measured using proton MR spectroscopy, were recently found to be associated with cognitive but not motor outcome in the same cohort [7].

In agreement with two recent studies [14], [15], WM ADC values did not contribute to the prediction of early outcome in our preterm cohort. Hence, we could not reproduce the finding of Krishnan and colleagues [16], who observed an inverse association between WM ADC on term DWI and outcome at two years’ CA among 38 preterm born infants without severe cerebral pathology. Neither WM nor cerebellar ADC values did relate to the presence of supra- or infratentorial brain injury as assessed using sequential cUS and TEA-MRI. Previous MRI studies have reported conflicting data on this topic.[10]–[13], [17] Diffusion parameters which take anisotropy into account may offer more insight in microstructural changes related to neonatal brain injury and secondary maturational disturbances [13].

The strength of the present study is that we used sequential cUS, TEA-MRI – performed within 24 hours of the term cUS examination – as well as supratentorial WM and cerebellar tissue DWI. There are, however, also several limitations that need to be addressed. Although the measured ADCs in our cohort demonstrated a high intra- and interobserver reliability, it is well known that these values are prone to partial volume effects due to inclusion of other tissue types, which may easily bias the data. Comprehensive sonographic evaluation of the cerebellum through the mastoid and posterior fontanel was not performed in all neonates. We therefore could not relate cerebellar cUS abnormalities to TEA-MRI, DWI and outcome. Regarding the limited additional prognostic value of T1- and T2-weighted TEA-MRI observed in this study, we have to acknowledge that assessment at two years’ CA may be too early to notice subtle deficits that may first manifest themselves at a later age [36].

In conclusion, these data support the clinical value of sequential cUS and demonstrate the benefits of an additional cUS at TEA. Cerebellar ADC values on term DWI may further contribute to prognostication in unselected preterm infants. Advanced research using more sophisticated MRI techniques may further improve the recognition of subtle brain pathology and provide more insight in preterm brain injury patterns related to the development of subsequent disabilities in childhood or adolescence.
